# Update to U.S. Medical Eligibility Criteria for Contraceptive Use, 2016: Updated Recommendations for the Use of Contraception Among Women at High Risk for HIV Infection

**DOI:** 10.15585/mmwr.mm6914a3

**Published:** 2020-04-10

**Authors:** Naomi K. Tepper, Kathryn M. Curtis, Shanna Cox, Maura K. Whiteman

**Affiliations:** 1 Division of Reproductive Health, National Center for Chronic Disease Prevention and Health Promotion, CDC.

“U.S. Medical Eligibility Criteria for Contraceptive Use” (U.S. MEC) 2016 provides evidence-based guidance for the safe use of contraceptive methods among U.S. women with certain characteristics or medical conditions (*1*). The U.S. MEC is adapted from global guidance from the World Health Organization (WHO) and kept up to date through continual review of published literature (*1*). CDC recently evaluated the evidence and the updated WHO guidance on the risk for human immunodeficiency virus (HIV) acquisition among women using hormonal contraception and intrauterine devices (IUDs) (*2*). After careful review, CDC adopted WHO’s 2019 updated guidance for inclusion in the U.S. MEC guidance; CDC’s updated guidance states that progestin-only injectable contraception (including depot medroxyprogesterone acetate [DMPA]) and IUDs (including levonorgestrel-releasing and copper-bearing) are safe for use without restriction among women at high risk for HIV infection (U.S. MEC category 1 [previously U.S. MEC category 2, advantages outweigh risks]) (Box). CDC’s guidance also adds an accompanying clarification for women who wish to use IUDs, which states “Many women at a high risk for HIV infection are also at risk for other sexually transmitted diseases (STDs). For these women, refer to the recommendations in the ‘U.S. Medical Eligibility Criteria for Contraceptive Use’ for women with other factors related to STDs, and the ‘U.S. Selected Practice Recommendations for Contraceptive Use’ on STD screening before IUD insertion” (*1*,*3*). Recommendations for other hormonal contraceptive methods (including combined hormonal methods, implants, and progestin-only pills) remain the same; there is also no restriction for their use among women at high risk for HIV infection (U.S. MEC category 1). Finally, CDC clarified that the U.S. MEC recommendations for concurrent use of hormonal contraceptives or IUDs and antiretroviral use for treatment of HIV infection also apply to use of antiretrovirals for prevention of HIV acquisition (preexposure prophylaxis [PrEP]).

BOX **Categories for classifying contraceptives**

1 = A condition for which there is no restriction for the use of the contraceptive method.

2 = A condition for which the advantages of using the method generally outweigh the theoretical or proven risks.

3 = A condition for which the theoretical or proven risks usually outweigh the advantages of using the method.

4 = A condition that represents an unacceptable health risk if the contraceptive method is used.

## Background

Although the rate of unintended pregnancy is decreasing in the United States, it remains high, with nearly half of pregnancies unintended (*4*). Increasing access to and promoting correct and consistent use of contraception is a priority strategy to reduce unintended pregnancies. HIV infection continues to be a major public health issue in the United States, with approximately 80% of new infections among women attributed to heterosexual contact (*5*). HIV infection is associated with adverse pregnancy outcomes for both the mother and child, including increased morbidity during pregnancy and perinatal HIV transmission (*6*). Therefore, prevention of both unintended pregnancy and HIV acquisition is critical among women at high risk for HIV infection.

Evidence on the potential association between contraceptive use and risk for HIV acquisition among women at high risk for HIV infection has been closely monitored by CDC and WHO. In July 2019, WHO held a consultation with external experts and stakeholders during which new evidence was reviewed. Following this consultation, WHO’s Guideline Development Group updated its recommendations to state that use of progestin-only injectable contraception, including DMPA, is MEC category 1 (safe for use without restriction) (*2*). In addition, WHO’s updated recommendations state that IUDs (both levonorgestrel-releasing and copper-bearing) are MEC category 1 with an accompanying clarification that women at high risk for HIV infection are also at risk for other STDs, and providers should refer to additional recommendations on IUD use for women at increased risk for STDs. Because of newly published studies and the WHO update, CDC initiated a process to assess whether its guidance should be updated for the U.S. context.

## Methods

CDC considered several factors and opinions in determining whether to update its guidance. First, CDC used two systematic reviews that were conducted preceding the 2019 WHO consultation: 1) an updated systematic review on hormonal contraception and risk for HIV infection to include new evidence since the last review (*7*), containing a total of 36 studies, 17 of which met minimum quality criteria defined in the review (*8*), and 2) a systematic review on copper IUD use and risk for HIV acquisition, containing seven studies, three of which met minimum quality criteria (*9*). The systematic reviews included primary reports of longitudinal studies (randomized clinical trials or observational studies) identified in PubMed or Embase databases through June 2019. Studies that met inclusion criteria compared incident HIV infection among women using hormonal contraception (injectables, oral contraceptives, implants, patches, rings, or hormonal intrauterine devices) or IUDs (copper or unspecified type) versus women using either 1) a nonhormonal method or no contraception or 2) a specific hormonal method of contraception. In these reviews, study quality was evaluated using a framework developed for a previous review on this topic (*7*) and updated to include quality criteria for randomized clinical trials.

In addition to the systematic reviews on contraception and risk for HIV acquisition, CDC considered the information on biologic mechanisms for HIV acquisition that was presented at the WHO consultation (*2*). CDC also reviewed existing reports on the epidemiology of unintended pregnancy, contraceptive use, maternal morbidity and mortality, and HIV infection in the United States as compared with the global context.

CDC also invited eight experts from outside the agency and one expert from within the agency to serve as ad hoc reviewers of the evidence and updated WHO recommendations. The reviewers were selected based on their expertise in HIV infection, family planning, or the intersection of these topics. The reviewers joined a teleconference with CDC staff members in September 2019 during which CDC staff members presented 1) the evidence; 2) the process and outcome of the updated WHO recommendations; and 3) the epidemiology of unintended pregnancy, contraceptive use, maternal morbidity and mortality, and HIV infection in the United States. The participants provided their individual input about 1) whether there has been a substantial evolution in the evidence regarding hormonal contraception or IUD use and HIV acquisition, 2) how the updated evidence might influence clinical practice in the United States, and 3) how the updated WHO recommendations might translate to clinical practice in the United States. Participants were not asked to develop recommendations or a consensus opinion. In addition, because PrEP is a key strategy in preventing HIV among women at high risk for HIV infection, CDC also considered whether the current U.S. MEC recommendations for concurrent use of hormonal contraceptives or IUDs and antiretroviral drugs for treatment of HIV also apply to use of antiretrovirals for PrEP.

After the teleconference, CDC developed the recommendations described in this report. CDC took into consideration the evidence, the updated WHO recommendations, and the individual perspectives provided by the expert reviewers.

## Rationale and Evidence

The primary source of new evidence was the Evidence for Contraceptive Options and HIV Outcomes (ECHO) trial, a large randomized clinical trial conducted in Eswatini, Kenya, South Africa, and Zambia that compared HIV acquisition among approximately 7,800 women using DMPA, levonorgestrel implants, or copper IUDs (*10*). No statistically significant differences in HIV acquisition were found among the three groups. This was deemed a high-quality study because of its large size, robust randomization and allocation procedures, high follow-up rates, high continuation of allocated contraceptive method, and comprehensive analysis (*2*). The design of the ECHO trial minimized the potential for confounding by sexual behavior and other factors that had limited the interpretation of previous observational studies.

Among the observational evidence examining the association between progestin-only injectables and risk for HIV acquisition, results were inconsistent across studies and limited by methodologic concerns (*8*). Limited evidence on other progestin-only contraceptives, combined hormonal contraceptives, and copper IUDs did not suggest increased risk for HIV acquisition, compared with other hormonal or nonhormonal contraceptives or no method (*8*,*9*).

Animal and laboratory data suggest possible biologic mechanisms for an association between hormonal contraceptive use and increased risk for HIV acquisition, including hormonally mediated changes in the vaginal epithelium and alterations in local and systemic immune responses. However, the relevance of these observations to clinical outcomes in women is unclear (*2*).

Although the rate of unintended pregnancy is declining, 45% of pregnancies in the United States were unintended in 2011, with higher percentages among women aged 15–19 years (75%) and black women (64%) (*4*). Contraceptive use and method distribution in the United States differs by certain characteristics, including age, race/ethnicity, and level of education (*11*). Although low overall, pregnancy-related mortality in the United States also differs significantly by race, with approximately a threefold higher risk among black compared with white women (*12*). In 2018, an estimated 7,100 newly diagnosed HIV infections occurred among U.S. women, with higher rates among racial/ethnic minorities (*5*). Although HIV prevalence is lower in the United States than in many areas globally (*13*), the risk for infection is higher among subgroups of women who have characteristics associated with nonuse of contraception, unintended pregnancy, and pregnancy-related complications (*5*).

PrEP is an important HIV prevention measure that is underutilized among women (*14*). Currently, daily oral PrEP with the fixed-dose combination of 300 mg of tenofovir disoproxil fumarate (TDF) and 200 mg of emtricitabine (FTC) has been shown to be safe and effective in reducing the risk for sexual HIV acquisition in adults and adolescents weighing at least 77 lbs (35 kg).* PrEP is recommended for HIV prevention for sexually active men and women reporting sexual behaviors that put them at substantial ongoing risk for HIV exposure and acquisition, and for men and women who inject drugs and report injection practices that put them at substantial ongoing risk for HIV exposure and acquisition (*15*). The U.S. MEC includes recommendations for safety and effectiveness of concurrent use of hormonal contraception or IUDs and antiretroviral drugs, based on a systematic review of the evidence on the potential for drug interactions between antiretroviral drugs and hormonal contraception (*1*,*16*). Studies of women who concurrently use PrEP and hormonal contraception have not demonstrated evidence of drug interactions (*16*).

## Recommendations for the Use of Hormonal Contraceptives and IUDs in Women at High Risk for HIV

CDC adopted the updated 2019 WHO guidance, which included changes to the recommendations for DMPA and IUDs. CDC’s updated recommendations are that all hormonal contraceptives (including implants, DMPA, progestin-only pills, and combined hormonal contraceptives) and IUDs (including levonorgestrel-releasing and copper-bearing) can be used without restriction among women at high risk for HIV infection (U.S. MEC category 1) (Table 1). For women using IUDs, a clarification to CDC’s updated recommendation states “Many women at a high risk for HIV are also at risk for STDs. For these women, refer to the recommendations in the ‘U.S. Medical Eligibility Criteria for Contraceptive Use’ for women with other factors related to STDs, and the ‘U.S. Selected Practice Recommendations for Contraceptive Use’ on STD screening before IUD insertion.”^†^ CDC also clarified that recommendations for use of hormonal contraceptives and IUDs among women using antiretroviral therapy apply to antiretroviral drug use for prevention (PrEP) or treatment of HIV (Table 2). These updated recommendations for the use of contraception among women at high risk for HIV infection assume that no other conditions are present; providers should consult the U.S. MEC to assess eligibility related to other medical conditions or characteristics (*1*).

**TABLE 1:** Updated recommendations for contraceptive use by women who are at high risk for human immunodeficiency virus (HIV) infection

Condition	Category								Clarifications/Evidence
	Cu-IUD		LNG-IUD		Implants	DMPA	POP	CHCs	
	I	C	I	C					
High risk for HIV	1	1	1	1	1	1	1	1	**Clarification (IUDs):** Many women at high risk for HIV are also at risk for other STDs. For these women, refer to the recommendations in the “U.S. Medical Eligibility Criteria for Contraceptive Use” for women with other factors related to STDs and the “U.S. Selected Practice Recommendations for Contraceptive Use” on STD screening before IUD insertion.*^,†^ **Evidence (IUDs):** High-quality evidence from one randomized clinical trial, along with low-quality evidence from two observational studies, suggested no increased risk for HIV acquisition with Cu-IUD use.^§^ No studies were identified for LNG-IUDs.^¶^ **Evidence (implants, DMPA, POP):** High-quality evidence from one randomized clinical trial observed no statistically significant differences in HIV acquisition between DMPA-IM versus Cu-IUD, DMPA-IM versus LNG implant, and Cu-IUD versus LNG implant.^¶,^** Of the low-to-moderate-quality evidence from 14 observational studies, some studies suggested a possible increased risk for HIV with progestin-only injectable use, which was most likely due to unmeasured confounding.^¶^ Low-quality evidence from 3 observational studies did not suggest an increased HIV risk for implant users.^¶^ No studies of sufficient quality were identified for POPs.^¶^ **Evidence (CHCs):** Low-to-moderate-quality evidence from 11 observational studies suggested no association between COC use (it was assumed that studies that did not specify oral contraceptive type examined mostly, if not exclusively, COC use) and HIV acquisition.^¶^ No studies of patch, ring, or combined injectable contraception were identified.^¶^

**Abbreviations:** C = continuation; CHC = combined hormonal contraceptive; COC = combined oral contraceptive; Cu = copper; DMPA = depot medroxyprogesterone acetate; I = initiation; IM = intramuscular; IUD = intrauterine device; LNG = levonorgestrel; POP = progestin-only pill; STD = sexually transmitted disease.

* Curtis KM, Tepper NK, Jatlaoui TC, Berry-Bibee E, Horton LG, Zapata LB, et al. U.S. medical eligibility criteria for contraceptive use, 2016. MMWR Recomm Rep 2016;65(No. RR-3).

^†^ Curtis KM, Jatlaoui TC, Tepper NK, et al. U.S. selected practice recommendations for contraceptive use, 2016. MMWR Recomm Rep 2016;65(No. RR-4).

^§^ Hannaford PC, Ti A, Chipato T, Curtis KM. Copper intrauterine device use and HIV acquisition in women: a systematic review. BMJ Sex Reprod Health 2020;46:17–25.

^¶^ Curtis KM, Hannaford PC, Rodriguez MI, Chipato T, Steyn PS, Kiarie JN. Hormonal contraception and HIV acquisition among women: an updated systematic review. BMJ Sex Reprod Health 2020;46:8–16.

** Evidence for Contraceptive Options and HIV Outcomes (ECHO) Trial Consortium. HIV incidence among women using intramuscular depot medroxyprogesterone acetate, a copper intrauterine device, or a levonorgestrel implant for contraception: a randomized, multicentre, open-label trial. Lancet 2019;394:303–13.

**TABLE 2:** Updated recommendations for contraceptive use by women who are using antiretrovirals*

Condition	Category								Clarifications/Evidence/Comments
	Cu-IUD		LNG-IUD		Implants	DMPA	POP	CHCs	
**Antiretrovirals used for prevention (PrEP) or treatment of HIV**	I	C	I	C					**Clarification (IUDs):** No known interaction exists between ARVs and IUDs. However, for women with HIV infection, IUD insertion is classified as category 2 if the woman is not clinically well or not receiving ARV therapy. Otherwise, both insertion and continuation are classified as category 1 (see HIV Infection section). For women at high risk for HIV, IUDs are category 1 for initiation and continuation (see High risk for HIV section). **Comment:** These recommendations generally are for ARV agents used alone. However, most women receiving ARVs are using multiple drugs in combination. In general, whether interactions between ARVs and hormonal contraceptives differ when ARVs are given alone or in combination is unknown.
**a. Nucleoside reverse transcriptase inhibitors (NRTIs)**									**Evidence:** NRTIs do not appear to have significant risk for interactions with hormonal contraceptive methods.†
i. Tenofovir (TDF) (Used for prevention [PrEP] or treatment)	1/2	1	1/2	1	1	1	1	1	
ii. Emtricitabine (FTC) (Used for prevention [PrEP] or treatment)	1/2	1	1/2	1	1	1	1	1	
iii. Zidovudine (AZT)	1/2	1	1/2	1	1	1	1	1	
iv. Lamivudine (3TC)	1/2	1	1/2	1	1	1	1	1	
v. Didanosine (DDI)	1/2	1	1/2	1	1	1	1	1	
vi. Abacavir (ABC)	1/2	1	1/2	1	1	1	1	1	
vii. Stavudine (D4T)	1/2	1	1/2	1	1	1	1	1	

**Abbreviations:** ARV = antiretroviral; C = continuation; CHC = combined hormonal contraceptive; COC = combined oral contraceptive; Cu = copper; DMPA = depot medroxyprogesterone acetate; HIV = human immunodeficiency virus; I = initiation; IUD = intrauterine device; LNG = levonorgestrel; POP = progestin-only pill; PrEP = preexposure prophylaxis.

* See full “U.S. Medical Eligibility Criteria for Contraceptive Use” for complete list of recommendations for all ARVs. No drug interactions between antiretroviral therapy and barrier methods are known. However, for spermicides and diaphragms (with spermicide), high risk for HIV is classified as category 4 because repeated and high-dose use of the spermicide nonoxynol-9 is associated with increased risk for genital lesions, which might increase the risk for HIV infection.

^†^ Nanda K, Stuart GS, Robinson J, Gray AL, Tepper NK, Gaffield ME. Drug interactions between hormonal contraceptives and antiretrovirals. AIDS 2017;31:917–52.

## Discussion

CDC adopted WHO’s guidance for inclusion in the U.S. MEC based on new, high-quality evidence that found the risk for HIV acquisition is similar across hormonal contraceptive methods and IUDs. Although the ECHO trial did not assess risk for HIV acquisition among nonusers, the trial addressed the relevant clinical question of differences in risk for HIV infection among contraceptive methods used by women desiring effective contraception. Women at high risk for HIV infection are eligible to use all hormonal contraceptive methods and IUDs without restriction. Although the context in the United States differs from the context globally in a number of ways (e.g., different contraceptive method mix, greater access to a range of contraceptive methods, lower risk for maternal morbidity and mortality, and generally lower HIV incidence), issues related to possible risks and the need for counseling are relevant across settings.

To avoid unintended pregnancy, access to the full range of safe and effective Food and Drug Administration–approved contraceptive methods is essential for all women, including those at high risk for HIV infection. Some additional considerations exist for use of barrier methods (*1*). Correct and consistent use of condoms can reduce the risk for pregnancy and acquisition of STDs, including HIV. No drug interactions between antiretroviral therapy and barrier methods are known. However, for spermicides and diaphragms (with spermicide), high risk for HIV is classified as category 4 (unacceptable health risk) because repeated and high-dose use of the spermicide nonoxynol-9 is associated with increased risk for genital lesions, which might increase the risk for HIV infection.

The rate of HIV acquisition in the ECHO trial was high overall (3.81 per 100 woman-years), despite participants receiving comprehensive HIV prevention services (*10*). HIV infection prevention measures should be strongly encouraged among all women at risk for HIV acquisition, including PrEP and postexposure prophylaxis, limiting numbers of sexual partners, and correct and consistent use of condoms.^§^ Family planning providers are in a unique position to offer HIV prevention services to women at high risk for HIV infection. Although STD (including HIV) counseling and screening services are not required for safe initiation of contraception (*3*), they are a core component of providing family planning and should be offered in accordance with CDC’s guidelines on STD treatment and HIV testing (*17*,*18*). Further integration of HIV prevention services, including PrEP, into family planning services could substantially increase access to these prevention measures for women at risk for HIV acquisition (*19*).

**Summary**

**What is already known about this topic?**

Prevention of both unintended pregnancy and human immunodeficiency virus (HIV) acquisition is critical among women at high risk for HIV infection.

**What is added by this report?**

CDC updated recommendations in the “U.S. Medical Eligibility Criteria for Contraceptive Use” to state that progestin-only injectable contraception (including depot medroxyprogesterone acetate) and intrauterine devices (including levonorgestrel-releasing and copper-bearing) are safe for use without restriction among women at high risk for HIV infection.

**What are the implications for public health practice?**

Women at high risk for HIV are eligible to use all hormonal contraceptive methods and intrauterine devices. Recommended HIV infection prevention measures, including preexposure and postexposure prophylaxis, limiting number of sexual partners, and correct and consistent use of condoms, should be strongly encouraged among all women at high risk for HIV acquisition and should be integrated into family planning services.

## Footnotes

* https://www.cdc.gov/hiv/guidelines/preventing.html.

† For women with other factors related to STDs, the benefits of IUD use outweigh the risks (U.S. MEC Category 2). If a woman has not been screened for STDs according to STD screening guidelines, screening can be performed at the time of insertion and insertion should not be delayed.

§ https://www.cdc.gov/hiv/basics/prevention.html.
